# Benzene-Poly-Carboxylic Acid Complex, a Novel Anti-Cancer Agent Induces Apoptosis in Human Breast Cancer Cells

**DOI:** 10.1371/journal.pone.0085156

**Published:** 2014-02-11

**Authors:** Fuad Fares, Naiel Azzam, Basem Fares, Stig Larsen, Steen Lindkaer-Jensen

**Affiliations:** 1 Department of Human Biology, Faculty of Natural Sciences, University of Haifa, and Department of Molecular Genetics, Carmel Medical Center, Haifa, Israel; 2 Department of Controlled Clinical Studies and Biostatistics, University of Life Science, Oslo, Norway; 3 Department of Surgery and Cancer, Hammersmith Hospital Campus, Imperial College, London, United Kingdom; Karolinska Institutet, Sweden

## Abstract

Some cases of breast cancer are composed of clones of hormonal-independent growing cells, which do not respond to therapy. In the present study, the effect of Benzene-Poly-Carboxylic Acid Complex (*BP-C1*) on growth of human breast-cancer cells was tested. *BP-C1* is a novel anti-cancer complex of benzene-poly-carboxylic acids with a very low concentration of cis-diammineplatinum (II) dichloride. Human breast cancer cells, MCF-7 and T47D, were used. Cell viability was detected by XTT assay and apoptosis was detected by Flow Cytometry and by annexin V/FITC/PI assay. Caspases were detected by western blot analysis and gene expression was measured by using the Applied Biosystems® TaqMan® Array Plates. The results showed that exposure of the cells to *BP-C1* for 48 h, significantly (P<0.001) reduced cell viability, induced apoptosis and activated caspase 8 and caspace 9. Moreover, gene expression experiments indicated that *BP-C1* increased the expression of pro-apoptotic genes (CASP8AP1, TNFRSF21, NFkB2, FADD, BCL10 and CASP8) and lowered the level of mRNA transcripts of inhibitory apoptotic genes (BCL2L11, BCL2L2 and XIAP. These findings may lead to the development of new therapeutic strategies for treatment of human cancer using *BP-C1* analog.

## Introduction

Breast cancer is the most common malignancy and the leading cause of death of women in western countries [1]–[3] . The risk factors for breast cancer include age, hormonal related factors, diet, radiation exposure, environmental factors and family history [2]. While most cases of breast cancers occur in women without a family history, about 10% of cases are found in women with mutations in *BRCA1* or *BRCA2* genes. Women with harmful mutations in either *BRCA1* or *BRCA2* have a risk of breast cancer that is about five times the normal risk, and a risk of ovarian cancer which is about ten to thirty times the normal risk [4]. *BRCA1* belongs to a class of genes known as tumor suppressors, which maintain genomic integrity to prevent uncontrolled proliferation. Researchers have identified more than 600 mutations in the *BRCA1* gene, many of which are associated with an increased risk of cancer [5]–[8]

Breast cancer is commonly treated by various combinations of surgery, radiation therapy, chemotherapy, and hormone therapy. Prognosis and selection of therapy may be influenced by the age and menopausal status of the patient, the stage of the disease, histologic and nuclear grade of the primary tumor, estrogen-receptor (ER) and progesterone-receptor (PR) status, measures of proliferative capacity, and HER2/neu gene amplification [8], [9].

Among the possible causes of cancer, damage or methylation of DNA and other cellular molecules, by reactive oxygen species (ROS), ranks high as a major culprit in the onset and development of the disease [10], [11]. These by-products of normal metabolism, which increase in cases of inflammation and following exposure to exogenous sources, can induce cancer-causing mutations, oxidize lipids and proteins, and alter signal transduction pathways that enhance cancer risk [12]–[15]. Experimental studies support the role of ROS in cancer, in part by showing that dietary antioxidants, as well as endogenous antioxidants which neutralize or trap ROS, act as cancer preventing agents [16], [17]. Human observational studies provide further support, showing, on one hand, that oxidant stress increases with the clinical progression of breast cancer [17] and, on the other hand, that a diet rich in antioxidant-containing foods reduces the risk of certain cancers [18]. New data, however, show that some dietary antioxidants may have potential as adjuvants in cancer therapy, by their ability to induce apoptosis [19]–[22].

There have been incredible advancements made in the treatment of breast cancer. As a result, the rate of deaths due to breast cancer has been in decline. Some cases are composed of clones of hormonal-independent growing cells, which do not respond to therapy. Therefore, alternative therapies are needed to induce death of cancer cells. In the present study, the effects of Benzene-Poly-Carboxylic Acid Complex (*BP-C1*), a novel anti-cancer agent, on growth of human breast-cancer cells and on gene expression were tested. Our data indicated that *BP-C1* induced apoptosis in human breast cancer cells through activation of caspases, increasing the expression of pro-apoptotic genes and reducing the expression of apoptotic inhibitory genes.

## Results

### Cell viability

**Figure 1:**
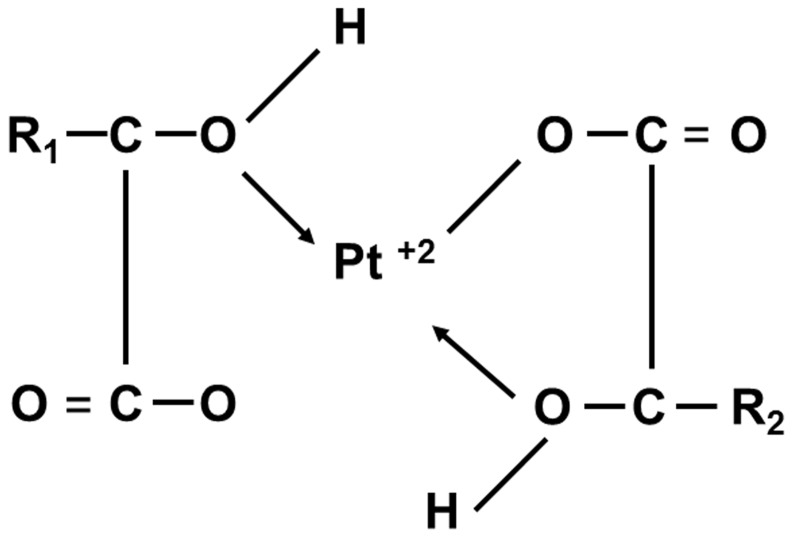
Chemical structure of BP-C1 analog. R1 and R2 represent any kind of chain.

**Figure 2:**
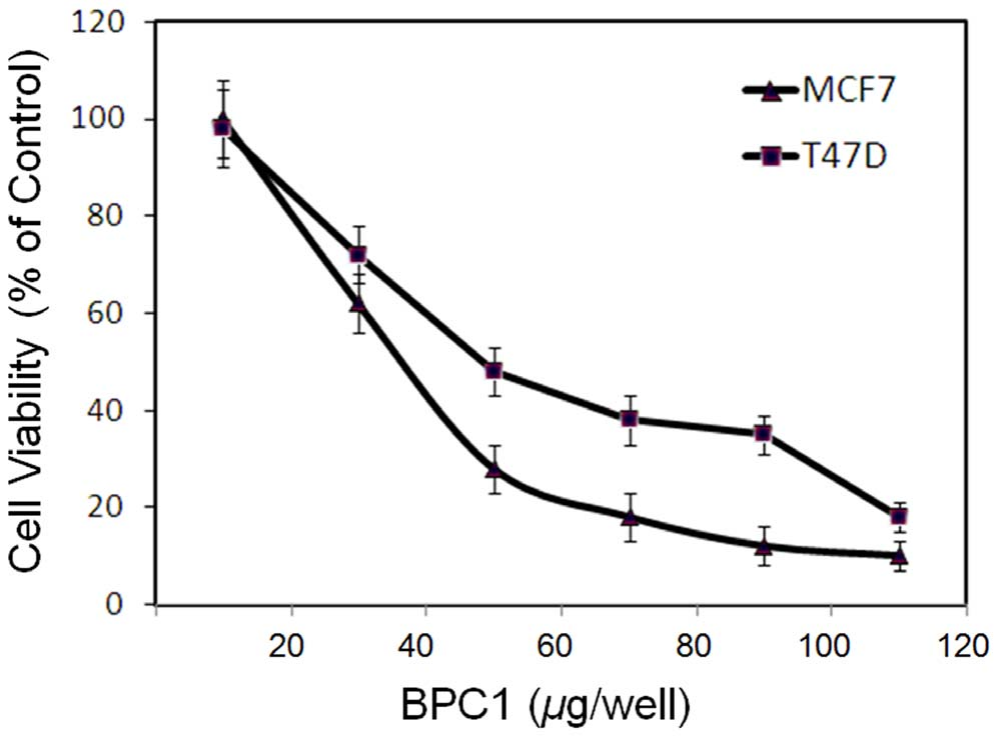
Dose - dependent growth inhibitory effect of *BP-C1* on MCF-7, T47D cells. Exponentially growing cells were incubated in the absence or presence of 100 to 1,000 µg/ml BP-C1 for 48 hours and the viable cells were counted as described under Materials and Methods. The results are presented as percentage of control and expressed as means ± standard deviation of three independent experiments in which each treatment was performed in triplicates.

In this study human breast cancer cell lines MCF-7 (wild-type p53) and T47D (p53 mutant) were used to examine the effects of *BP-C1* (Fig. 1) on cell proliferation. Cells were treated with BP-C1 (100–1,000 µg/ml) for 48 hours and cell viability was detected by XTT assay. The results indicated that *BP-C1* significantly (P<0.001) reduced cell viabilty of MCF7 and T47D cells with IC_50_ of 370 µg/ml and 490 µg/ml, respectively (Figure 2). In order to exclude the possibility of cytotoxic effects of *BP-C1* on the cells, LDH assay was performed as described under “Materials and Methods”. LDH assay is one of the most widely-used and accepted methods for measuring cellular lysis. It was observed that *BP-C1*, at a concentration of up to 1,500 µg/ml, does not cause a statistically significant change in the LDH level in the media compared with controls (Data not shown). Therefore, for further studies, 750 µg/ml, was used.

### Cell cycle analysis

In order to study the effect of *BP-C1* on cell cycle progression, cells were incubated with 750 µg/ml for 48 h, and the distribution of the cells in the different phases of cell cycle was determined by FACS as described under “Materials and Methods”. Treatment of the cells with *BP-C1* resulted in a reduction in G1 phase and an accomulation of cells at the sub-G1 phase (Table 1). The distribution of MCF7 and T47D cells in G1 phase in control vs treatment were 50±4% vs 16±2%, and 59±5% vs 19±3% respectively. On the other hand, an increase of cells in the sub-G1 phase was observed after *BP-C1* treatment, compared to control in both cell lines, (MCF-7 60±6% vs 16±2%; T47D 56±3% vs 13±1%), suggesting an occurance of cell death in the sub-G1 phase.

**Table 1:** The effect of *BP-C1* on cell cycle progression.

		Sub – G1 Phase (%)	G1 Phase (%)	S Phase (%)	G2/M Phase (%)
	**Control**	16±2	50±4	16±2	15±4
**MCF7 Cells**	**750 µg/ml**	60±6	16±2	10±2	8±1
	**Control**	13±1	59±5	13±2	14±2
**T47D Cells**	**750 µg/ml**	56±3	19±3	8±1	10±1

MCF-7 cells were treated with 750 µg/ml for 48 h. followed by treatment with RNase (100 µg/ml) and DNA staining with propidium iodide (PI). Cell cycle was analyzed using the FACSCalibur Cell Sorter.

### Detection of apoptotic cells

**Figure 3:**
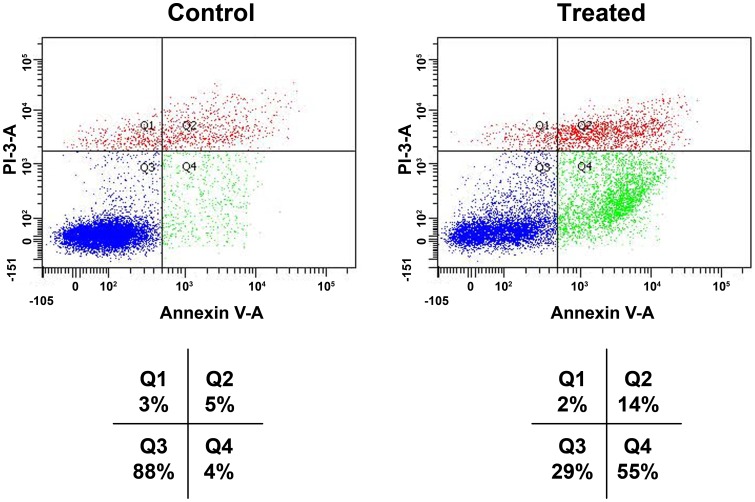
Effects of *BP-C1* on annexin V level on MCF-7 CELLS. Cells were treated with *BP-C1* (750 µg/ml) for 48 h and Flow cytometric analysis of annexin V-FITC/PI double-stained was performed. In each plot, the lower left quadrant (Q3) represents viable cells, the upper left quadrant (Q1) indicates necrotic cells, the lower right quadrant (Q4) denotes early apoptotic cells, and the upper right quadrant (Q2) represents necrotic or late apoptotic cells.

**Figure 4:**
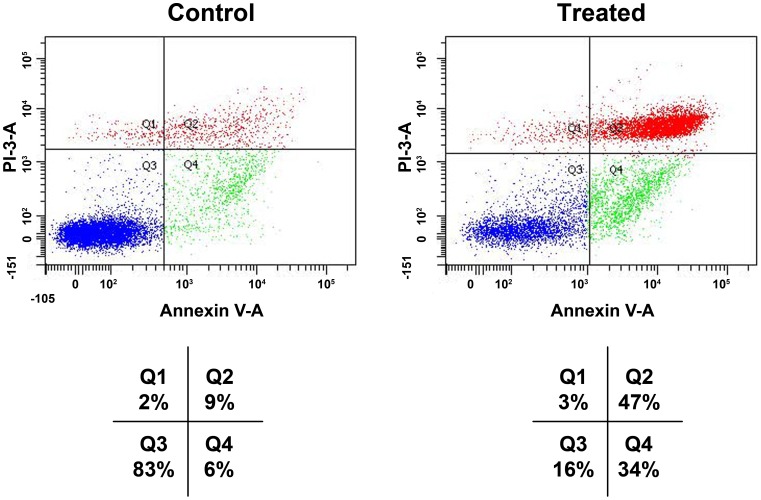
Effects of *BP-C1* on annexin V level on T47D CELLS. Cells were treated with *BP-C1* (750 µg/ml) for 48 h and Flow cytometric analysis of annexin V-FITC/PI double-stained was performed. In each plot, the lower left quadrant (Q3) represents viable cells, the upper left quadrant (Q1) indicates necrotic cells, the lower right quadrant (Q4) denotes early apoptotic cells, and the upper right quadrant (Q2) represents necrotic or late apoptotic cells.

Annexin V-FITC binding analysis and PI staining were performed to quantify cell death arising from apoptosis and necrosis, respectively. The number of intact cells was significantly decreased in the treated cells with *BP-C1* compared to the control cells as shown in Fig. 3 and Fig. 4. Treatment with *BP-C1* significantly increased (P<0.05) the number of Annexin V-FITC-positive/PI-positive cells. Respective percentages of these cells (Q2+Q4) after treatment with 750 µg/ml for 48 h were: 69% vs. 9%, for MCF-7 cells (Fig. 3) and 81% vs. 15% of T47D cells (Fig. 4).

### Effect of *BP-C1* on caspase activation

**Figure 5:**
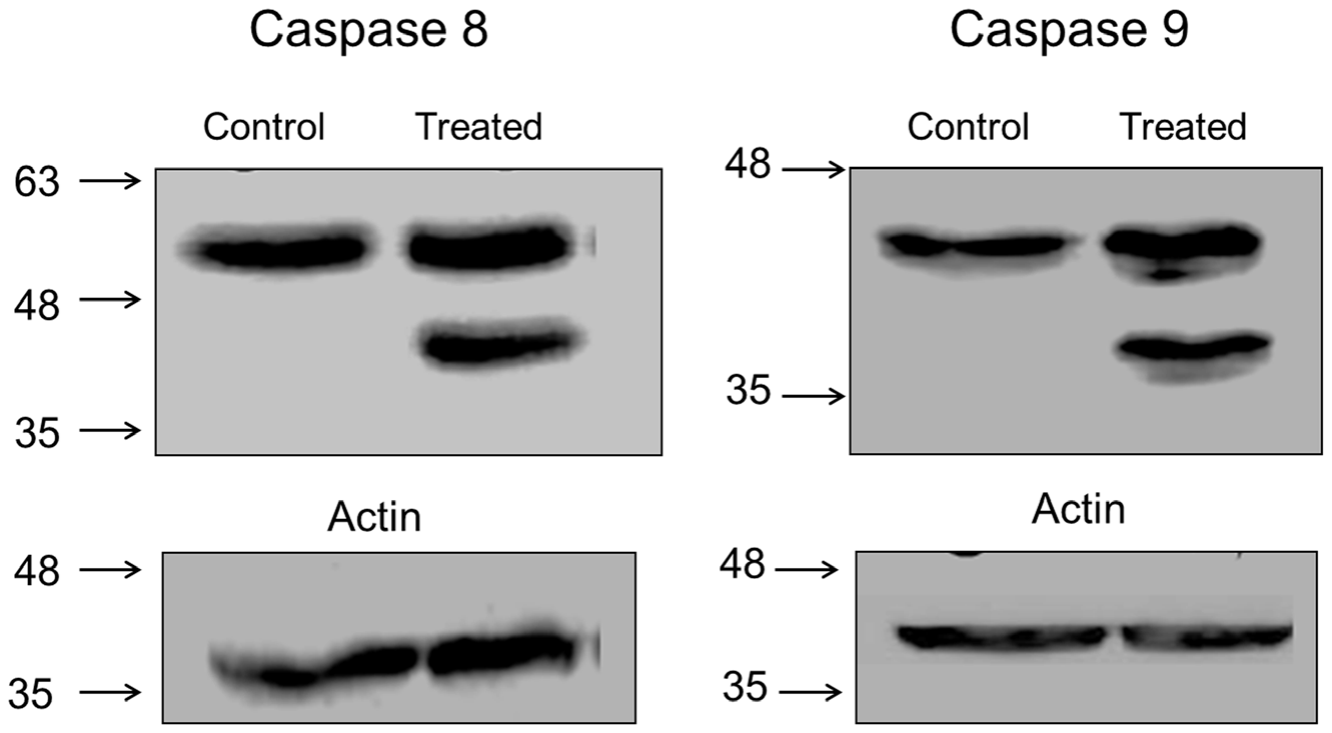
Activation of caspase 8 (A) and caspase 9 (C) by *BP-C1*. Cells were treated with or without 750/ml of BP-C1 for 48 h. Cells were harvested and lysed. Activation of caspase-9 was determined by Western blotting as described under “Materials of Methods”. TA represents paclitaxel and 32c refers to Lx2-32c. Columns represent the densitometry analysis of the indicated proteins. The level of actin in the cells is represented in B and D.

To further investigate the mechanistic action of cell death induced by *BP-C1*, a western blot analysis was performed to detect proteins that have been shown to be involved in both the extrinsic (caspase 8) and intrinsic (caspase 9) apoptosis pathways (Fig. 5). Cells were treated with an effective dose of *BP-C1*(750 µg/ml), lysed and subjected to western blot analysis for cleaved caspases 8 (Fig. 5A) and 9 (Fig. 5C) after 48 h of treatment, results indicate the appearance of the cleaved active subunit of caspase 8 (43 kDa) and caspase 9 (35 kDa), however the level of actin did not changed (Fig. 5. B and D).

### Effect of BP-C1 on gene expression

**Figure 6:**
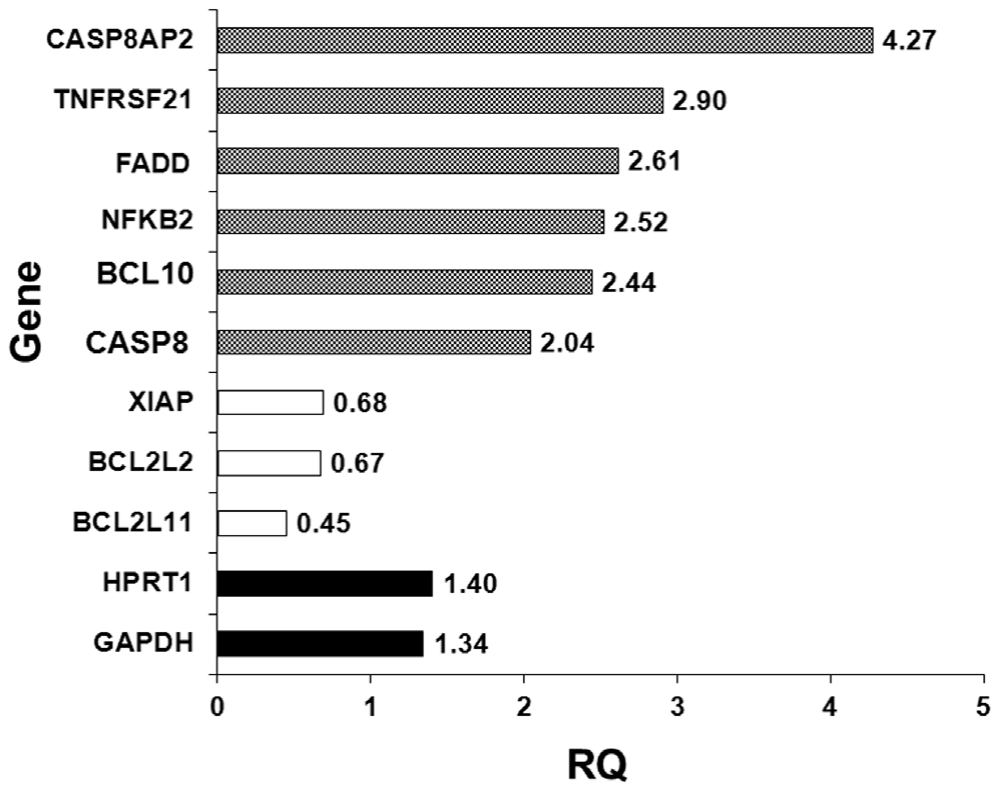
The effect of *BP-C1* on gene expression. MCF-7 cells were treated with 750 µg/ml for 48 h. After treatment, total RNA was isolated and reverse transcribed.Gene expression of pro-apoptotic genes was detected using the Applied Biosystems® TaqMan® Array Plates. HPRT1 and GAPDH genes were used as housekeeping genes for internal control to correct the potential variation in RNA loading.

The effect of *BP-C1* on the expression of genes involved in apoptosis was detected using the Applied Biosystems® TaqMan® Array–KIT Gene expression quantified by qRT-PCR as described under “Materials and Methods”. The results revealed higher levels of mRNA transcripts of pro-apoptotic genes; CASP8AP2 (RQ:4.27), TNFRSF21 (RQ:2.90), FADD (RQ:2.61), NFKB2 (RQ:2.52), BCL10 (RQ:2.44), CASPAS8 (RQ:2.41) when compared to the expression level of housekeeping genes; HPRT1 (RQ:1.40) and GAPDH (RQ:1.34). On the other hand, gene expression quantified by qRT-PCR revealed lower levels of mRNA transcripts of apoptotic-inhibitory genes; BCL2L11 (RQ: 0.45), BCL2L2 (RQ: 0.67) and XIAP (RQ:0.68) (Fig. 6).

## Discussion

The present study has shown that the novel anticancer agent, *BP-C1*, is significantly reduced cell viability of human breast cancer cells through induction of apoptosis via activation of caspase 8 and caspase 9, increasing the expression of pro-apoptotic genes and reducing the expression of apoptotic inhibitory genes . Furthermore, *BP-C1* has no effect on release LDH into the cell medium, providing evidence that *BP-C1* seems to be an untoxic agent. *BP-C1* is a combination of benzo-poly-carbonic acid and platinum in a very low concentration. Benzo-poly-carbonic acid is a polymer complex of carbonic and oxicarbonic acids rich in carboxyl-groups replacing the chlorine ions. Through reaction with platinum, a cis-configuration diamino-dicarboxilate complex is formed. These carboxyl groups, being a part of a complex organic compound, are bound to platinum more tightly than the chlorine ions, and this has a positive impact on the toxicity of the compound compared to cisplatin and carboplatin.

*BP-C1* is thought to act in the same way as platinum compounds in general, which are known to be able to react with DNA to form intra - and interstrand crosslink. Cisplatin cross links DNA in several different ways, interfering with cell division by mitosis. The damaged DNA elicits DNA repair mechanisms, which in turn activate apoptosis when repair proves impossible. It was shown that the apoptosis induced by cisplatin on human colon cancer cells depends on the mitochondrial serine-protease Omi/Htra2 [23]. On the other hand, any mechanisms of cisplatin resistance have been proposed including changes in cellular uptake and efflux of the drug, increased detoxification of the drug, inhibition of apoptosis and increased DNA repair [24]. Oxaliplatin is active in highly cisplatin-resistant cancer cells in the laboratory; however, there is little evidence for its activity in the clinical treatment of patients with cisplatin-resistant cancer. The drug paclitaxel may be useful in the treatment of cisplatin-resistant cancer; the mechanism for this activity is unknown [25]. Moreover, Cisplatin has a number of side-effects that can limit its use such as; Nephrotoxicity (kidney damage), Neurotoxicity (nerve damage), Ototoxicity (hearing loss): Electrolyte disturbance: Cisplatin can cause hypomagnesaemia, hypokalaemia and hypocalcaemia and Hemolytic anemia [26]–[29].

The toxicity and efficacy of *BP-C1* are investigated in a non-randomised multi-centre Phase I dose - escalating trial with fifteen Stage IV breast cancer patients using a 3-level Response Surface Pathway design. *BP-C1* is given intramuscularly once daily during 32 days with a cumulative starting dose of 0.64 mg/kg body weight. Priliminary results indicated that BP-C1 has no side effects and it is safe for use (Unpublished data).

On this background, the present mechanism of action study was performed to evaluate how *BP-C1* affects cancer cell growth on the molecular and cellular basis. The present study provides evidence that *BP-C1* induces apoptosis in high (MCF-7 cells) as well as in low differentiated (T47D cells) human breast cancer cells. BP-C1 activates the initiator caspases, caspase 8 and 9. Caspases are regulated at a post-translational level ensuring that they can be rapidly activated. They are first synthesized as inactive pro-caspases that consist of a prodomain, a small subunit and a large subunit. The prodomain of the initiator caspases contain domains such as a CARD domain (e.g., caspases-2 and -9) or a death effectors' domain (DED) (caspases-8 and -10) that enables the caspases to interact with other molecules that regulate their activation [30].

Gene expression expeiment indicated that BP-C1 increased the expression of proapoptotic genes (CASP8AP2, TNFRSF21, FADD, NFKB2, BCL10 and CASPAS8) and reduced the expression of apoptotic inhibitory gene (BCL2 family genes and XIAP). CASP8AP2 is highly similar to FLASH, a mouse apoptotic protein identified by its interaction with the death-effectors' domain (DED) of caspase 8. Studies of FLASH protein suggested that this protein may be a component of the death-inducing signaling complex that includes Fas receptor, Fas-binding adapter FADD and caspase 8, and plays a regulatory role in Fas-mediated apoptosis [31], [32]. Tumor necrosis factor receptor superfamily member 21 (TNFRSF21), also known as death receptor 6 (DR6). The protein encoded by this gene is a member of the TNF-receptor super family. This receptor has been shown to activate NF-κB and MAPK8/JNK, and induce cell apoptosis. Through its death domain, this receptor interacts with TRADD protein, which is known to serve as an adaptor that mediates signal transduction of TNF-receptors [33]. Fas-associated death domain protein (FADD) is an adaptor molecule that bridges the interactions between membrane death receptors and initiator caspases. Thus, the site of its action has always been expected to be the cytoplasmic death-inducing signaling complex (DISC) [34], [35]. Activation of NF-kB has been linked to inflammatory events and inhibition of NF-kB has been linked to apoptosis and delayed cell growth. The finding that NF-kB is activated immediately before apoptosis has led to suggestion that this transcreption factor may function to promote apoptosis [36]–[38]. B-cell lymphoma/leukemia 10 (BCL10), this gene was identified by its translocation in a case of mucosa-associated lymphoid tissue (MALT) lymphoma. The protein encoded by this gene contains a caspase recruitment domain (CARD), and has been shown to induce apoptosis and to activate NF-kappaB [39]. CASP8 gene encodes a member of the cysteine-aspartic acid protease (caspase) family. Sequential activation of caspases plays a central role in the execution-phase of cell apoptosis. This protein is involved in the programmed cell death induced by Fas and various apoptotic stimuli. The N-terminal FADD-like death effector domain of this protein suggests that it may interact with Fas-interacting protein FADD [40].

The results also indicated that BP-C1 reduced the expression of anti-apoptotic genes. The protein encoded by this gene belongs to the BCL-2 protein family. BCL-2 family members form hetero- or homodimers and act as anti- or pro-apoptotic regulators that are involved in a wide variety of cellular activities. The protein encoded by this gene contains a Bcl-2 homology domain 3 (BH3) [41], [42]. Bcl-2-like protein 2 is a protein that in humans is encoded by the *BCL2L2* gene [42]. This gene encodes a pro-survival (anti-apoptotic) member of the bcl-2 protein family. The proteins of this family, form hetero- or homodimers, act as anti- and pro-apoptotic regulators. Expression of this gene in cells has been shown to contribute to reduced cell apoptosis under cytotoxic conditions. Studies of the related gene in mice indicated a role in the survival of NGF- and BDNF-dependent neurons. Mutation and knockout studies of the mouse gene demonstrated an essential role in adult spermatogenesis. X-linked inhibitor of apoptosis protein (XIAP), also known as inhibitor of apoptosis protein 3 (IAP3) and baculoviral IAP repeat-containing protein 4 (BIRC), is a protein that in humans is encoded by the XIAP gene [43], [44]. XIAP also known as inhibitor of apoptosis protein 3 (IAP3) and baculoviral IAP repeat-containing protein 4 (BIRC), is a protein that in humans is encoded by the XIAP gene.

Gene expression studies indicated that *BP-C1* activates genes that contain guanine-adenine (GA) repeats in their promotor region. Therefore, it is hypothesised that *BP-C1* acts as GA-binding proteins (GABP) which are *ets* transcription factors that control gene expression in several important biological settings. *Ets* factors are intimately involved in critical cellular functions, including development, cellular differentiation, apoptosis, and carcinogenesis [45], [46].

In conclusion, the results of the present study may contribute to a better understanding of the molecular mechanisms by which *BP-C1* exerts its effect on human breast cancer cells and tumours in general. *BP-C1* may induces apoptosis in different pathways such as the extrinsic and the intrinsic pathways. These findings may lead to the development of new strategies for the treatment of human breast cancer in advanced stages, for which there is at present no effective life-prolonging therapy.

## Materials and Methods

### Chemical and Biological Reagents

Cell culture media and reagents were purchased from Biological Industries (Beit Haemek, Israel). *BP-C1* was purchased from Meabco, Copenhagen, Denmark.*BP-C1* is a benzene-poly-carboxylic acids complex with cis-diammineplatinum [II] dichloride. This is a polymer complex of carbonic and oxicarbonic acids rich in carboxyl-groups replacing the chlorine ions. Through reaction with platinum, a cis-configuration diamino-dicarboxilate complex is formed. These carboxyl groups, being a part of a complex organic compound, are bound to platinum more tightly than the chlorine ions, and have a positive impact on toxicity of the compound compared to cisplatin and carboplatin. One *ml* of *BP-C1* contains 0.5 mg of platinum-ammonium salts of benzene-poly-carbonic acids, including 0.05 mg of platinum (Fig. 1).

Anti – caspase 8 monoclonal antibody was purchased from Oncogene (Boston, MA, USA). Anti caspase 9 monoclonal antibody was purchased from Medical and Biological Laboratoties (Japan). Anti actin monoclonal antibodies was purchased from INC Biochemicals (Aurora, OH, USA). Secondary antibody peroxidase – conjugated goat anti – mouse IgG was purchased from Jackson Immune Research Laboratories (West Grove, PA, USA). All other chemicals were purchased from Sigma (Saint Louis, MO, USA) or other local sources.

### Cell Culture

The human breast cancer cell lines: MCF-7 (wild-type p53) and T47D (mutant p53) were purchased from American Tissue Culture Collection (ATCC, Bethesda, MD). Cells were cultured at 37°C in a humidified 5% CO_2_ atmosphere in DMEM medium supplemented with 100 U/ml penicillin, 100 mg/ml streptomycin, 2 mM of L-glutamine and 10% fetal calf serum (Biological Industries, Beit Haemek, Israel). To the growing media of MCF7 cells, 0.25 U/ml of insulin was added.

### Cell Viability

MCF-7 and T47D cells were seeded in 96-well plates (2×10^4^ and 2×10^3^ cells/well). The following day, cells were treated with BP-C1 in a spectrum of concentrations ranging from 100 to 1,000 µg/ml for 48 hours and cell viability was detected using Cell Proliferation Assay, XTT (Biological Industries, Beit Haemek, Israel). This assay is based on the ability of metabolic active living cells to reduce the tetrazolium salt, XTT, to orange colored compounds of formazan. The absorbance of each sample was measured using an ELISA reader (Tecan, Spectra) at a wavelength of 450 nm with a reference absorbance of 620 nm. The results are presented as percentage of control and expressed as means ± standard deviation of three independent experiments in which each treatment was performed in triplicates.

### Lactate dehydrogenase (LDH) release

Cellular damage, such as necrosis, causes an elevation of the LDH concentration in the medium. The integrity of the plasma membrane following treatment was determined by measuring LDH activity released into the culture medium. The enzyme activity was measured using a spectrophotometric method [47].

### Cell Cycle Analysis

Cells were trypsinized, fixed in 70% ethanol and stored at 4°C until a FACS analysis was conducted. The ethanol was removed and the cells were incubated with NP-40 0.1% on ice for 5 min followed by a washing with cold PBS and treatment with RNase (100 µg/ml) for 1 h. For DNA staining, propidium iodide (PI) was added to the cells at a concentration of 50 µg/ml, while keeping them at 4°C for 20 min. Cells were analyzed using the FACSCalibur Cell Sorter (Becton Dickinson, NJ, USA).

### Annexin V-FITC/PI double-staining assay

Cell death was further analyzed by double staining the cells with FITC-labeled Annexin V and propedium iodide (PI), using an Annexin V-FITC apoptosis detection kit, (BD Biosciences, San Jose, CA, USA), according to the manufacturer's instructions. Briefly, both floating and adherent cells were collected, resuspended in an Annexin V binding buffer and transferred to test tubes containing FITC-labeled Annexin V and PI. The cells were then incubated for 15 minutes at room temperature in dark, and analyzed by flow cytometry using the FACS Calibur system (BD Biosciences, San Jose, CA, USA). Annexin V-FITC and PI emissions were detected in the FL 1 and FL 2 channels, using 525 and 575 nm emission filters, respectively. The Annexin V-FITC-negative/PI-negative population was considered to include all normal healthy cells. Annexin V-FITC-positive/PI-negative cells were regarded as a measure of early apoptosis. The Annexin V-FITC-positive/PI-positive population was considered to represent late apoptotic or necrotic cells, and the Annexin V-FITC-negative/PI-positive cells were considered to include necrotic cells. The percentage distributions of normal, early apoptotic, late apoptotic, and necrotic cells were calculated using ModFitLT V3.0 software (BD Biosciences, San Jose, CA, USA).

### Western blot analysis

Proteins were extracted, using a RIPA buffer (1% NP-40, 0.5% sodium deoxycholate, 0.1% SDS, 1 mM EDTA) containing protease inhibitors (30 µg/ml aprotinin, 2.5 µg/ml leupeptin, 10 µg/ml pepstatin and 1 mM phenylmethyl fluoride) as previously described [48].

Samples were electrophorised on denaturing 15% SDS-polyacrylamide gels as described above [49]. Gels were allowed to equilibrate for 10 min in 25 mM Tris and 192 mM glycine in 20% (vol/vol) methanol. Proteins were transferred to a 0.2 µm pore size nitrocellulose membrane (Sigma, Saint Louis, MO, USA) at 250 mA for 3 h, using a Mini Trans-Blot electrophoresis cell (Biorad Laboratories, Richmond, CA, USA) according to the method described in the manual accompanying the unit. The nitrocellulose membrane was blocked with 5% non-fat dry milk for 2 h at room temperature and incubated with caspase 8 or caspase 9 antibodies (1∶1,000 titer) for overnight at 4°C. Three consecutive washes in PBS containing 0.1% Tween (10 min/wash) were performed and the membrane was incubated with a secondary antibody conjugated to Horse Radish Peroxidase (HRP) (Zymed, San Francisco, CA, USA) for 2 h at room temperature. The nitrocellulose paper was washed three times and finally, was reacted with enhanced chemiluminescent substrate (ECL) (Pierce, Rockford, IL USA) for 5 min, dried with Whatman sheet and exposed to X-ray film.

### Gene expression analysis

Gene expression was detected using the Applied Biosystems® TaqMan® Apotosis Array Plate for human apoptosis (Cat.No 4414072, Applied Biosystems, Foster city, CA). The panel of genes in this plate contains 92 Taqman assays for apoptosis associated genes and 4 Taqman assays of endogenous control genes. This panel targets genes from both, the signaling pathways that initiate mammalian apoptosis, the death receptor regulated pathway, and the BCL-2 family pathway, together with few caspases involved in the final mechanisms of cell death. After treatment, total RNA was isolated using the TRIzol reagent (Invitrogen, Carlsbad, CA). 1 µg of RNA was reverse transcribed using a reverse transcription system according to the manufacturer's instruction. Then , quantitative real-time PCR amplification was performed using Taqman array plate, to which the synthesized cDNA was added. Each well contains sequence specific primers for known gene and dye-labeled MGB probe, that enables detection of amount of cDNA real time amplification of each targeted gene in the plate. Housekeeping genes, HPRT1 and GAPDH, were used for internal control to correct the potential variation in RNA loading. All reactions were performed on the STEPONEPLUS Real-Time PCR system (Applied Biosystems) and in a 10 µl volume containing the cDNA sample, TaqMan fast universal PCR mastermix, primers, and probes. Before the PCR cycles, samples were incubated for 2 min at 50°C and for 10 min at 95°C. Thermal cycles consisted of 40 cycles at 95°C for 5 sec and 60°C for 30 sec.

The results were analyzed using the DataAssist™ Software, version 3 (supplied by Applied Biosystems, Foster city, CA), which enables rapid and comprehensive interpretation of TaqMan® Array Plate results. DataAssist™ Software provides a filtering procedure for outlier removal, various normalization methods based on single or multiple genes and relative quantification (RQ) analysis of gene expression through a combination of statistical analysis and interactive visualization. DataAssist™ Software is freely available at http://www.appliedbiosystems.com/DataAssist.

### Statistical Analysis

Each experiment was repeated at least three times and results were expressed as the mean ± SEM. Statistical analysis of the data were performed using Student's *t* test and ANOVA. P<0.05 was considered significant.
